# Differential modulation of behavior by infraslow activities of different brain regions

**DOI:** 10.7717/peerj.12875

**Published:** 2022-02-01

**Authors:** Duho Sihn, Sung-Phil Kim

**Affiliations:** Department of Biomedical Engineering, Ulsan National Institute of Science and Technology, Ulsan, Republic of Korea

**Keywords:** Infraslow activity, Electroencephalogram, Behavior modulation, Behavioral performance, Brain region, Oscillation phase

## Abstract

The oscillation phase of electroencephalograms (EEGs) is associated with behavioral performance. Several studies have demonstrated this association for relatively fast oscillations (>1 Hz); a similar finding has also been reported for slower oscillations, showing that behavioral performance is correlated with the phase of infraslow activity (ISA, 0.01–0.1 Hz) of electroencephalography (EEG). However, the previous study only investigated ISA in a local brain region using a relatively simple task (somatosensory discrimination task), leaving it difficult to determine how the EEG ISA for various brain regions is associated with behavioral performance. In addition, it is not known whether the EEG ISA phase modulates more complex behavioral task performance. In the present study, we analyzed the ISA of whole-brain EEG of participants performing various behaviors while playing video games. We found that behavior was associated with the specific oscillation phase of EEG ISA when that behavior was independent of other behaviors. In addition, we found that the EEG ISA oscillation phases modulating the different behaviors varied across brain regions. Our results suggest that the EEG ISA for different brain regions modulates behavioral performance in different ways and such modulation of EEG ISA can be generalized to diverse behaviors. This study may deepen the understanding of how EEG ISA modulates behavior and increases the applicability of EEG ISA.

## Introduction

Brain activity oscillates at various frequencies. Several studies have shown that the phase of relatively fast oscillations (>1 Hz) is associated with behavior. For instance, the phase of alpha or theta oscillations, as measured by electroencephalography (EEG), is associated with the performance of visual perception tasks (Busch, Dubois & VanRullen, 2009; Dugué, Marque & VanRullen, 2011; Hanslmayr et al., 2013; Spaak, De Lange & Jensen, 2014) and that of delta oscillations or other stimulus-entrained oscillations is associated with the performance of auditory perception tasks (Henry, Herrmann & Obleser, 2014; Herbst & Obleser, 2019). In addition, the attentional level is modulated by the phase of theta oscillations (Helfrich et al., 2018).

However, behavior often fluctuates over a long period. Long-range temporal correlations among brain oscillations are related to behaviors such as decision-making (Colosio et al., 2017; Nakao et al., 2019; Sugimura et al., 2021). On the one hand, very slow oscillations can accommodate the time required for a behavior that varies over a long-range temporal scale. Monto et al. (2008) showed that the oscillation phase of EEG infraslow activity (ISA, 0.01–0.1 Hz) is correlated with behavioral performance, which also fluctuates at an infraslow frequency. In particular, they showed that behavioral performance increased during the rising phase of the EEG ISA. However, this study used a relatively simple somatosensory discrimination task and measured the local ISA using only two EEG electrodes (Fz and Cz). It is therefore unclear whether the oscillation phase of EEG ISAs in different brain regions is associated with different behavior.

There have been other studies on EEG ISA in humans. However, these studies investigated EEG ISA during sleep (Marshall et al., 2000; Vanhatalo et al., 2004; Lázár, Dijk & Lázár, 2019) or rest without behavioral tasks (Karavaev et al., 2018; De Goede & Van Putten, 2019). Therefore, to the best of our knowledge, no study has extended the findings of Monto et al. (2008) to other behavioral tasks. It is well documented that different types of behavior are associated with relatively fast brain oscillations over different brain regions. For instance, emotion is associated with alpha oscillations (8–13 Hz) in frontal region (Coan & Allen, 2004), visual spatial attention is associated with alpha oscillations (7–14 Hz) in occipital region (Sauseng et al., 2005), focused internal attention is associated with alpha oscillations (8–12 Hz) in right parietal region (Benedek et al., 2014), and object processing is associated with beta oscillations (13–18 Hz) in temporo-parietal region (Von Stein et al., 1999), to name a few. These ample findings indicate a possibility that slow brain oscillations occurring in different brain regions would also be related to different cognitive processes. If this is the case, the performance of cognitive functions spanning relatively long-term durations (*e.g.*, tens of seconds) such as sustained attention or decision making can be associated with the phase of infraslow oscillations. This would offer a unique opportunity to find neural correlates of the performance of various types of behavior in relatively slow temporal windows. Since various types of behavior may be associated with EEG ISAs in different brain regions, it would be imperative to investigate modulations of behavior with EEG ISAs over different brain regions.

Thus, the present study aimed to address the following two questions regarding EEG ISA: (1) Do EEG ISAs in different brain regions modulate behavior differently? (2) Can EEG ISA modulation be extended to various types of behavior? To this end, we analyzed the whole-brain EEG of participants exhibiting various behaviors while playing a video game. Specifically, we focused on six types of behavior performed during the gameplay (“Missile launch button”, “Collect star”, “Collect ammo box”, “Crash into wall”, “Crash into enemy”, and “Hit enemy by missile”) and examined the association of each behavior with the oscillation phase of EEG ISA. We also analyzed these associations in different brain regions.

## Materials & Methods

### Dataset

We used the publicly available dataset downloaded from https://openneuro.org/datasets/ds003517/versions/1.1.0 (Cavanagh & Castellanos, 2021). The dataset contains two behavioral tasks: “Exemplar tasks” and “Escape from Asteroid Axon”. We only used data of the “Escape from Asteroid Axon” behavioral task (video game). A 63-channel EEG (500-Hz sampling rate) was recorded when 17 participants played the video game. The experiment on the “Escape from Asteroid Axon” behavioral task lasted 45 min. The player in the video game aimed to shoot missiles to hit enemies while avoiding obstacles (walls and enemies). The player’s six behaviors were logged as follows: “Missile launch button” (the player shoots missiles.), “Collect star” (The player fills the health bar.), “Collect ammo box” (The player fills the missiles.), “Crash into wall” (The player hits the wall. Then, the health bar decreases.), “Crash into enemy” (The player hits the enemy. Then, the health bar decreases.), and “Missile hit enemy” (Missiles fired by the player hit the enemy.) Participants’ behaviors were logged using a Logitech F310 gamepad (Cavanagh & Castellanos, 2016).

Monto et al. (2008) used a somatosensory discrimination task which needs perceptual sensitivity. To extend these findings to other brain functions, we chose this “Escape from Asteroid Axon” task which needs a function of visuo-motor control (“Missile launch button”, “Crash into wall”, “Crash into enemy”, and “Missile hit enemy”) and an overall sustained attention to a behavioral task (“Collect star” and “Collect ammo box”). The purpose of this study was to investigate whether these behaviors, which are known to be related to different brain functions, are modulated by EEG ISA in the same or different brain regions.

### Behavioral data analysis

Since the six behaviors could be closely related to each other in playing the game, we analyzed the dependencies of the different behaviors. One type of these dependencies is the causal relationship between behaviors: If a missile fired by the player hits the enemy (“Missile hit enemy”), the player must have fired the missile before that (“Missile launch button”). Another type of dependencies to consider is probabilistic antecedents: When the player fires a missile (“Missile launch button”), the missile won’t always hit the enemy (“Missile hit enemy”), but it will hit the enemy with a certain probability (“Missile hit enemy”). In this sense, “Missile launch button” is a probabilistic antecedent behavior of “Missile hit enemy”. The reverse is also possible: If the player continues to fire multiple missiles, the player may have continued to fire missiles (“Missile launch button”) after the first of those missiles hit the enemy (“Missile hit enemy”). In this case, “Missile hit enemy” is a probabilistic antecedent behavior of “Missile launch button”. As another example, if the player hits a wall and the health bar is reduced (“Crash into wall”), the player will try to fill the health bar by collecting stars (“Collect star”). In this case, “Crash into wall” is a probabilistic antecedent behavior of “Collect star”. We measured these probabilistic antecedents that reflect the behavioral task structure. The reason for measuring these probabilistic antecedents is that if there is a strong antecedent relationship between the behaviors, the timing of occurrence of an ensuing behavior is likely to be mainly modulated by the antecedent behavior, not the EEG ISA. If there is no strong antecedent relationship between behaviors, our hypothesis is that the behaviors will be modulated by EEG ISA.

**Figure 1:**
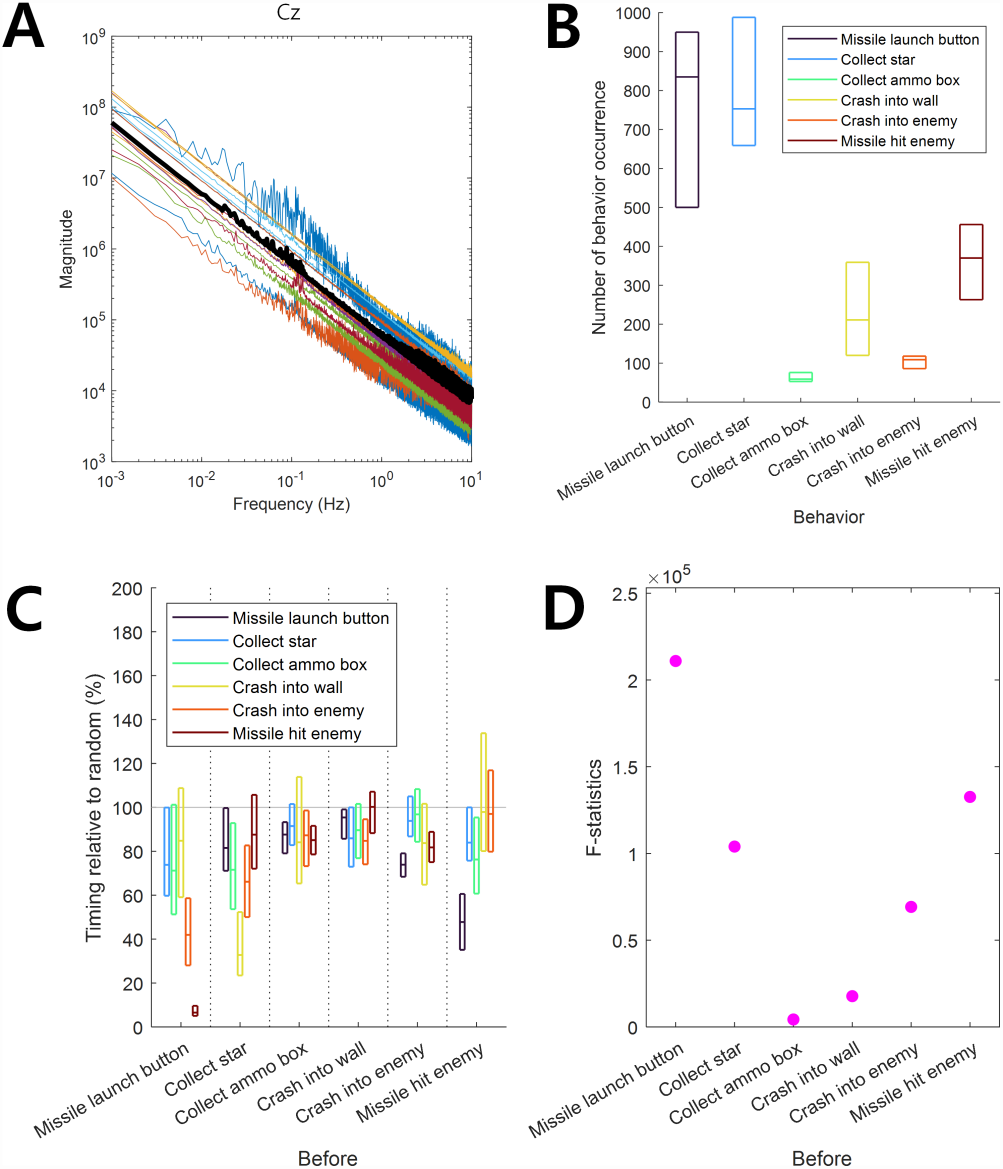
Infraslow EEG and behaviors. (A) Frequency components of EEG. Color-coded curves indicate the frequency-magnitude curve for each participant. The thick black curve indicates the median. (B) The behavior occurrence frequency for each behavior. The horizontal lines in each box indicate 25%, 50%, and 75% of data, respectively. (C) The behavior occurrence dependency for other behaviors was quantified by the timing relative to random (See ‘Materials and Methods’ section). The horizontal lines in each box indicate 25%, 50%, and 75% of data, respectively. The gray horizontal line at 100% indicates the random level. (D) *F*-statistics of data in C.

We quantified how a behavior (*e.g.*, Missile hit enemy) depended on the preceding occurrence of another behavior (*e.g.*, Missile launch button) by calculating the ratio of the actual to the pseudorandom durations after the onset of the preceding behavior before the onset of behavior 1. We called this quantification as “the timing relative to random” (see Fig. 1C).

Specifically, let *t* be the time of occurrence of behavior 1 and let *s*_*t*_ be the duration after the onset of the preceding behavior before the onset of behavior 1. For simplicity, we downsampled the timing data for the occurrence of all behaviors from 500 Hz to 1 Hz. Let *r*_*t*_ be the pseudo-random latest occurrence timing of the preceding behavior before the occurrence of behavior 1. *r*_*t*_ was obtained from the sequence of Poisson random numbers generated once per second. The mean parameter of the Poisson random numbers was estimated from the actual frequency of the preceding behavior. Let $ \left\langle {r}_{t} \right\rangle $ be the average of the 10,000-independently generated *r*_*t*_’s. The timing relative to the random is given by:

(1) \begin{eqnarray*}\text{Timing relative to random}=100{ \left\langle \frac{{s}_{t}}{ \left\langle {r}_{t} \right\rangle } \right\rangle }_{1}\end{eqnarray*}

where ${ \left\langle \cdot \right\rangle }_{1}$ is an average over all occurrences of behavior 1.

To quantify how the occurrence of one behavior depends on the occurrence of another behavior, we assessed how random the closeness of the dependency (timing relative to random; Eq. (1)) was (100%). If the timing relative to random was markedly below 100%, it indicated that the occurrence of behavior 1 was dependent on the occurrence of the preceding behavior.

We also determined whether the dependencies of some behaviors were more significant than those of others. To quantify this, we measured how much the dependency on the preceding behavior (timing relative to random) differed for each of five preceding behaviors. We expressed this as the ratio of the variability of the dependencies between preceding behaviors to the variability of the dependencies within each preceding behavior, which is the *F*-statistics as a quantification indicator. For each behavior, we calculated the timing relative to random values for the behavior and the other five behaviors for each participant using Eq. (1). We calculated the *F*-statistics of the timing relative to random among the five behaviors. A high *F*-statistic (*i.e.,* a large difference in the timing relative to random for the other five behaviors) indicated that the corresponding behavior was highly dependent on the other specific behavior(s), while a low *F*-statistic indicated that the corresponding behavior was less dependent on the occurrence of specific behaviors.

### EEG ISA analysis

To determine whether the EEG data showed specific frequency peaks in the infraslow frequency band, which would indicate that there was a dominant oscillation in the ISA, the magnitude for each of the frequencies of the EEG data was computed. The magnitude of raw EEG data at each frequency was measured by the absolute value of the Fourier transformed EEG data, which was computed by the fast Fourier transform (FFT) algorithm (see Fig. 1A).

To extract ISA from the raw EEG data, we performed bandpass filtering at 0.01–0.1 Hz. We also applied the Hilbert transform to estimate the oscillation phase of the ISA. Note that the oscillation phase at 0 rad was located at the maximum amplitude of the ISA, and the oscillation phase at −*π* = *π* rad was located at the minimum. The oscillation phase at $ \left[ -\pi ,0 \right] $ rad was the rising phase, and the oscillation phase at $ \left[ 0,\pi \right] $ rad was the falling phase. After the Hilbert transform, we downsampled the EEG ISA phase data from 500 Hz to 1 Hz to place the phase data at the same time scale as the behavioral data (see above).

To investigate the association between behavior and EEG ISA, the ISA phase was divided into eight oscillation phase bins: *i* = 1, …, 8. The central phase of each bin was $ \left( 2i-1 \right) \pi /8$ and the width of each bin was *π*/4. The occurrence of each behavior in each participant was counted for each phase bin: *b*_*i*_, *i* = 1, …, 8. The normalized occurrence of a behavior with respect to the ISA phase is calculated as

(2) \begin{eqnarray*}{n}_{i}= \frac{{b}_{i}/{p}_{i}}{\sum _{j=1}^{8}{b}_{j}/{p}_{j}} \end{eqnarray*}

where *p*_*i*_ is the measure of the oscillation phase duration for each phase bin *i* = 1, …, 8. Instead of assuming that the oscillation phases of the EEG ISA occur equally in all phase bins, we normalized to the amount that actually occurs.

**Figure 2:**
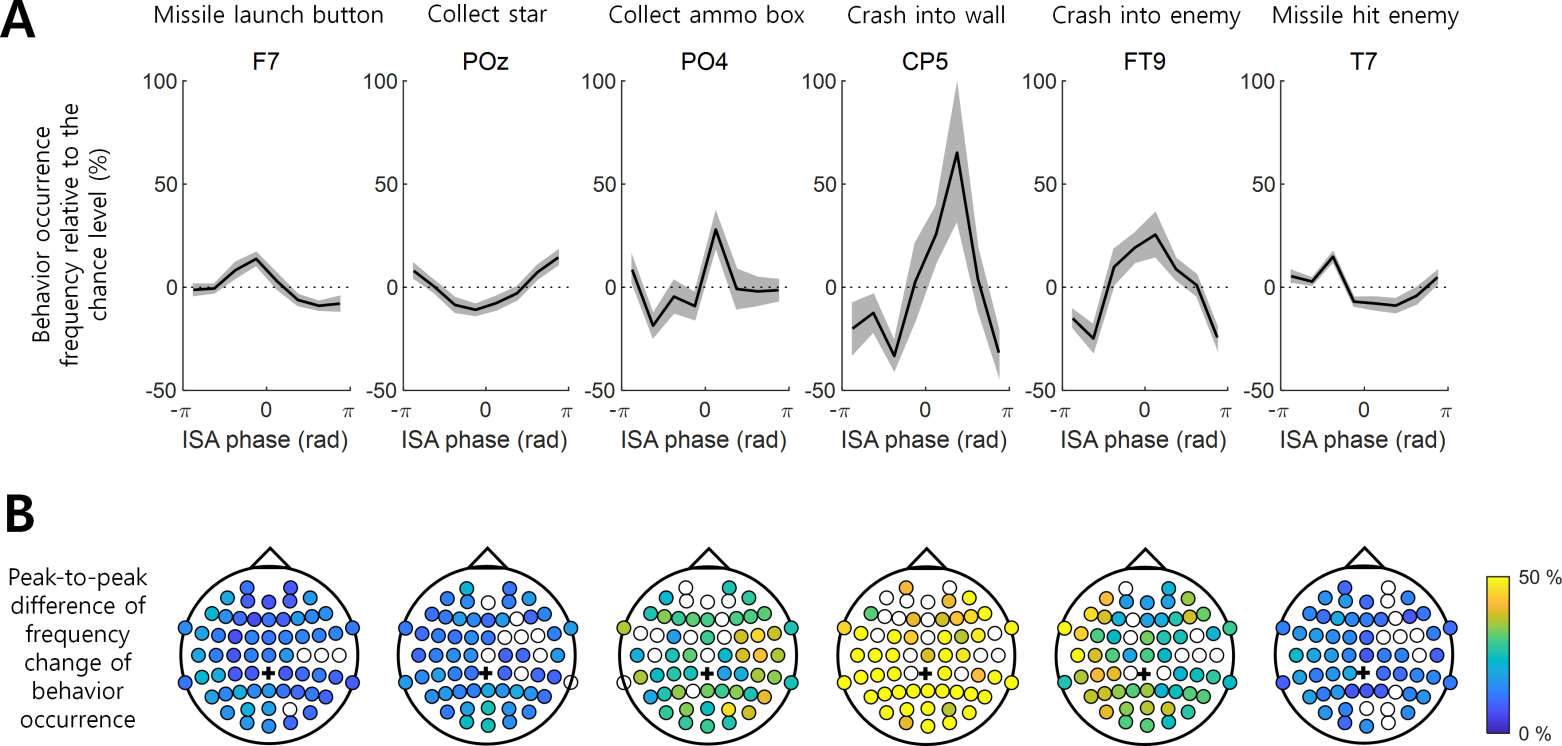
Behavior occurrence frequency on EEG ISA oscillation phase. (A) The behavior occurrence frequency relative to the chance level (See ‘Materials and Method’). Each channel was selected if it showed the largest peak-to-through difference. Bold black line indicates the mean for the participants. Gray shading area indicates the standard error of the mean. (B) The peak-to-trough difference of the frequency change in the behavior occurrence (See ‘Materials and Method’; *d*_peak-to-trough_). The filled circle indicates the EEG channel with the statistical significance between the max. occurrence oscillation phase and min. occurrence oscillation phase (*t*-test, *p* < 0.05, *N* = 17). The empty circle represents the EEG channel with no statistical significance. Black cross indicates the reference channel.

To quantify how significantly the occurrence of a behavior varied with the EEG ISA phase, the behavior occurrence frequency relative to the chance level, *m*_*i*_, at an oscillation phase bin *i* (see Fig. 2A), was computed as follows:

(3) \begin{eqnarray*}{m}_{i}=100 \left( 8{n}_{i}-1 \right) .\end{eqnarray*}

Note that because ${\mathop{\sum }\nolimits }_{i=1}^{8}{n}_{i}=1$ and ${\mathop{\sum }\nolimits }_{i=1}^{8}{m}_{i}=0$, Eq. (3) indicates the zero-centered deviation of behavioral occurrence on the percentile scale, if *n*_*i*_ = 1/8 (chance level), *m*_*i*_ = 0, and a positive (or negative) value of *m*_*i*_ indicates a more (or less) frequent occurrence of behavior *i*.

These *m*_*i*_ values for Eq. (3) were first obtained for each participant and averaged over all the participants. This behavior occurrence frequency relative to the chance level (*m*_*i*_) was the basis for further analyses after averaging for all participants. The peak-to-trough difference in the behavior occurrence frequency over the phase bins, *d*_peak-to-trough_, was computed as follows:

(4) \begin{eqnarray*}{d}_{\text{peak-to-trough}}={m}_{\alpha }-{m}_{\beta }\end{eqnarray*}

where *α* = argmax_*i*_*m*_*i*_ and *β* = argmin_*i*_*m*_*i*_. To identify the EEG channels that showed more significant behavioral modulation, a normalized peak-to-trough difference in behavior occurrence over the phase bins was obtained by normalizing *d*_peak-to-trough_ over all the EEG channels. We used MATLAB for all our analyses.

## Results

### Dependencies among behaviors

The frequency-magnitude curve of EEG showed log–log linearity without a clear peak at the 0.01–0.1 Hz infraslow frequency band (Fig. 1A). This result was consistent with that of a previous study by Monto et al. (2008). The frequency-magnitude curve in Fig. 1A was reminiscent of 1/f noise, giving rise to the doubt that slow EEG may be simply composed of noise with no brain activity. However, as we demonstrated below, the ISA of EEG data used in this study revealed associations with behavior, indicating that ISA at 0.01–0.1 Hz carried information related to brain activity, which was not illustrated in the frequency-magnitude curve.

The frequency of occurrence of each behavior during game play was different. Among the six behaviors (“Missile launch button”, “Collect star”, “Collect ammo box”, “Crash into wall”, “Crash into enemy”, and “Missile hit enemy”), the occurrence frequencies of “Missile launch button” and “Collect star” were relatively high whereas those of “Collect ammo box” and “Crash into enemy” were relatively low (Fig. 1B).

We observed dependencies among behaviors such that the occurrence of some behaviors depended on the preceding occurrence of others. The timing relative to random of “Missile launch button” before the occurrence of the behaviors of “Crash into enemy” and “Hit enemy by missile” was significantly lower than 100% (*t*-test, *p* = 10^−8^, *N* = 17), showing that the “Missile launch button” behavior was likely to precede the other two behaviors (Fig. 1C). Similarly, “Missile launch button” occurred after “Missile hit enemy”, “Collect star” occurred after “Crash into wall”, and “Missile hit enemy” occurred after “Missile launch button”. On the other hand, the times of the occurrences of “Collect ammo box”, “Crash into wall”, and “Crash into enemy” were independent of the occurrence of other behaviors, showing a timing relative to random close to 100% (Fig. 1C) and lower *F*-statistic values (Fig. 1D, see Methods for the interpretation of *F*-statistics).

### Different behaviors are associated with the EEG ISA phases for different brain regions

The degree to which the occurrence of a behavior was modulated by the ISA phase and the phase at which the occurrence frequency was maximized varied with the behaviors and EEG channel locations. For instance, the variation in the occurrence frequency (*m*_*i*_ in Eq. (3) in Methods) of “Crash into wall” was most pronounced at channel CP5, where this behavior occurred most frequently at the ISA phase of $ \left[ \pi /4,2\pi /4 \right] $ rad with the max.–min. occurrence difference (*d*_peak-to-troug_) of 98.51%. In contrast, the min. occurrence difference (*d*_peak-to-troug_) for “Missile launch button” was 22.73% and maximized at channel F7, showing the most frequent occurrence at $ \left[ -\pi /4,0 \right] $ rad (Fig. 2A). The representative modulation of the occurrence frequency of other behaviors by the ISA phase is also depicted in Fig. 2.

Overall, a stronger modulation by the ISA phase was observed for the “Collect ammo box”, “Crash into wall”, and “Crash into enemy” behaviors relative to others (Fig. 2). It is noteworthy that these three behaviors were less dependent on the occurrence of other behaviors (Fig. 1) and showed stronger associations with the phase of EEG ISA than the other three behaviors more dependent on other behaviors.

**Figure 3:**
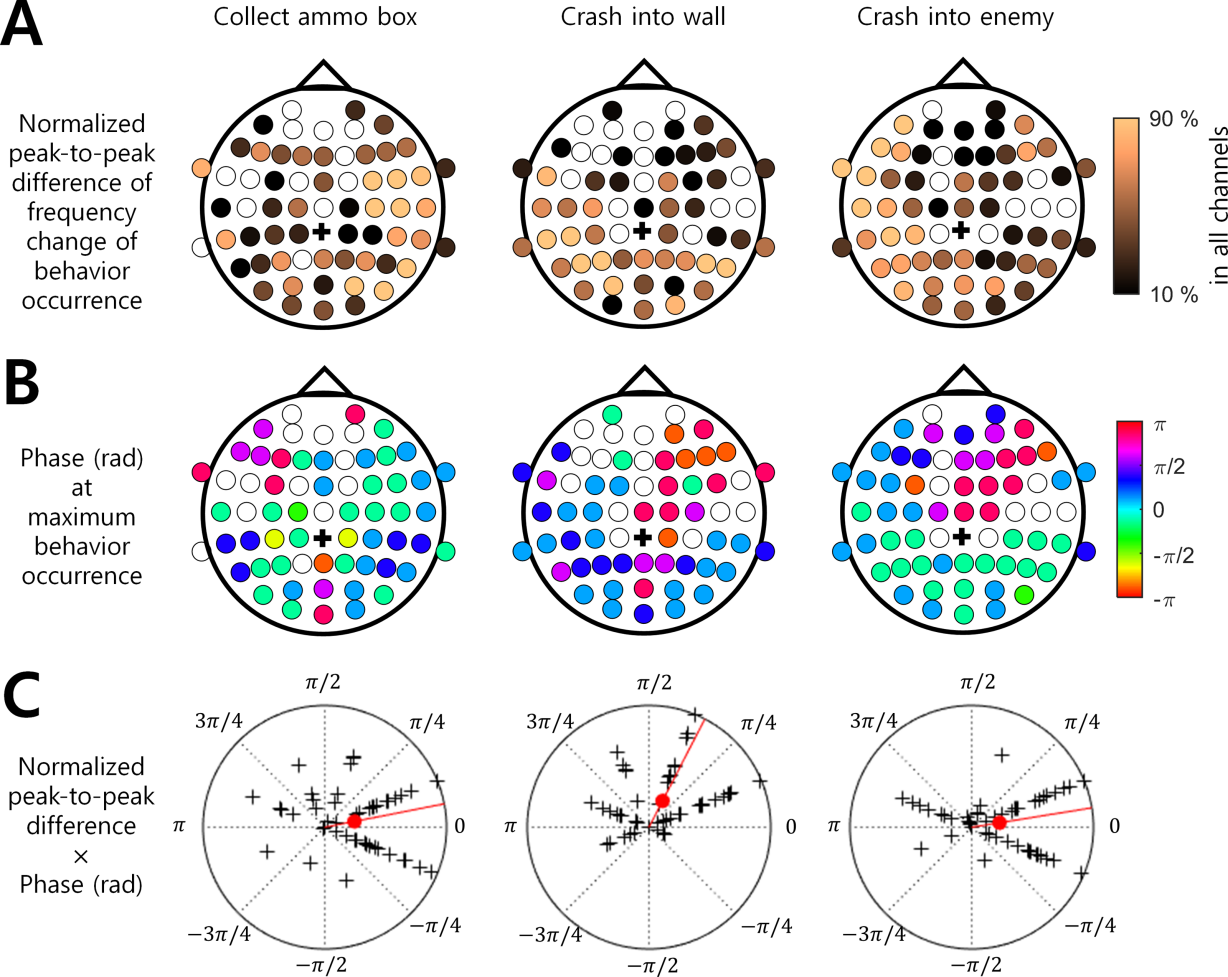
Brain region and EEG ISA oscillation phase with the significant behavior occurrence. (A) Normalized peak-to-trough difference in the frequency change of behavioral occurrence (See ‘Materials and Method’). (B) Phase (rad) at maximum behavior occurrence (See ‘Materials and Method’). The filled circle indicates the EEG channel with the statistical significance between the max. occurrence oscillation phase and min. occurrence oscillation phase (*t*-test, *p* < 0.05, *N* = 17). The empty circle indicates the EEG channel with no statistical significance. The black cross indicates the reference channel. (C) The normalized peak-to-trough difference × Phase (rad) at maximum behavior occurrence. The magnitude of the polar plot indicates the normalized peak-to-trough difference in the frequency change of behavioral occurrence. The phase of the polar plot indicates the phase (rad) at maximum behavior occurrence. The black cross indicates a channel. The filled red circle indicates the circular mean over all channels. The red line indicates the phase of the circular mean.

Among the three behaviors that showed stronger associations with the ISA phase, “Collect ammo box” was more likely to occur at the maximum phase (*i.e.,* around 0 rad) in the right hemispheric regions. In addition, “Crash into wall” was more likely to occur at the falling ISA phase over the left posterior region, and “Crash into enemy” was more likely to occur at the maximum ISA phase over the left hemispheric region (Figs. 3A and 3B). To represent this tendency of the ISA phase modulation for each behavior, we created a vector of a phase at which the behavior occurred most frequently (Fig. 3B) and scaled the length of the vector by normalized peak-to-trough difference (Fig. 3A). In other words, the angle of the phase vector of a channel is the oscillation phase at maximum behavior occurrence and the magnitude of the phase vector of a channel is the normalized peak-to-through difference of that channel. We collected the phase vectors from all channels (marked by black crosses in Fig. 3C) and calculated the vector sum which was the mean resultant vector. The angle of this vector sum (marked by the red line in Fig. 3C) indicates the dominant phase at which each behavior is modulated. It demonstrated again that “Crash into wall” was mainly modulated at the falling phase whereas the other two behaviors were mainly modulated at the maximum phase (Fig. 3C).

## Discussion

In a previous study, Monto et al. (2008) showed that the behavioral performance of the somatosensory discrimination task was increased during the rising phase of EEG ISA measured at the Fz and Cz channels. In the present study, we extended the results of Monto et al. (2008) by using more complicated behaviors and inspecting a wider area of the brain. Specifically, we analyzed EEG ISA for six behaviors while playing a video game. We found that if the occurrence of a behavior (*e.g.*, crash into wall) did not depend on other behaviors (Fig. 1), the occurrence of this behavior was more strongly associated with the specific oscillation phase of EEG ISA (Fig. 2). This suggests that relatively simple perceptual behaviors and more diverse and complex behaviors can be modulated by EEG ISA. We also found that different behaviors were associated with different EEG ISA oscillation phases in different brain regions (Fig. 3). This suggests that the association of the EEG ISA with a certain behavior can be specific with respect to both the brain region and phase.

The timing of occurrence of “Missile launch button”, “Collect star”, and “Missile hit enemy” depended on the occurrence of “Missile hit enemy”, “Crash into wall”, and “Missile launch button”, respectively (Fig. 1). “Missile launch button” is a probabilistic antecedent of “Missile hit enemy”; When a missile is fired, it has a certain probability to hit the enemy. “Missile hit enemy” is a probabilistic antecedent of “Missile launch button”; If the missiles are fired in succession, the missiles are fired even after the missiles fired at the beginning hit the enemy. “Crash into wall” is a probabilistic antecedent of “Collect star”; If the health bar shrinks due to crashing a wall, the player must collect stars to refill the health bar. These are because of the task structure. Although those behaviors (“Missile launch button”, “Collect star”, and “Missile hit enemy”) are affected by specific brain functions, the timing of their occurrence is not significantly modulated by the EEG ISAs because the timing of their occurrence is highly dependent on the occurrence of other behaviors (Fig. 2). On the other hand, the timing of occurrence of “Collect ammo box”, “Crash into wall”, and “Crash into enemy” did not depend on the occurrence of other behaviors (Fig. 1). The timing of occurrence of these behaviors was well modulated by EEG ISAs (Fig. 2). This suggests the possibility that EEG ISA can modulate behavior in a general fashion because all behaviors with little dependence on other behaviors appeared to be strongly modulated by ISA in the present study.

In relatively fast oscillations, it is known that different types of behavior are associated with different brain regions. This is related to the localization of brain function. Therefore, we hypothesized that EEG ISAs in different brain regions would modulate different behaviors. Since different behaviors in the game require different brain functions, we thought that different brain regions would be involved.

EEG ISAs in different brain regions were associated with different behaviors (Figs. 2 and 3). First, the behavior of “Collect ammo box” was modulated by EEG ISAs in the right hemisphere. “Collect ammo box” can be deemed to be associated with continuously paying attention to the remaining number of missiles. Such sustained attention is known to be associated with neural activity in the right hemisphere (Pardo, Fox & Raichle, 1991). Thus, it is likely that EEG ISAs in the right hemisphere reflecting sustained attention might be associated with the behavior of collecting ammo box. Second, the behavior of “Crash into wall” was mainly modulated by EEG ISAs in the left posterior region. “Crash into wall” represents a failure in the integration of visual input and motor control. The posterior brain region is known to be responsible for field-limb integration in the visually guided reaching task (Kertzman et al., 1997). As such, more occurrences of crashing into wall would be related to deficits in parietal visuo-motor integration. In addition, the fact that all participants controlled the game with the right hand might suggest that left parietal regions were more relevant to the failure of visuo-motor integration leading to crashing into wall. Third, the behavior of “Crash into enemy” was modulated by the left hemisphere. Similar to “Crash into wall”, “Crash into enemy” can be viewed as a failure in integrating visual input and movement control, but unlike “Crash into wall”, this includes the element of destroying the enemy. By giving meaning to actions, it might have reinforced “Crash into enemy”. As the left hemisphere is known to be involved in the interpretation of action (Gazzaniga, 1989), EEG ISA in the left hemisphere might be related to the feedback of motor outcomes for destroying the enemy as well as the failure of visuomotor integration.

“Missile launch button” and “Missile hit enemy” can represent good behavioral performance, which occurred most frequently at the rising oscillation phase of ISA (Fig. 2). On the other hand, “Crash into wall” could represent poor behavioral performance, which occurred most frequently in the falling oscillation phase, that is, the converse of the rising oscillation phase (Fig. 3). These results were consistent with the previous result by Monto et al. (2008), in that the behavioral performance was maximized at the rising oscillation phase.

In the present study, the frequency–magnitude curve showed log–log linearity in the infraslow frequency band (Fig. 1A). Although this can be reminiscent of 1/f noise, we posit that it could be quasiperiodic, as indicated by the log–log linearity rather than the 1/f noise (Palva & Palva, 2012; Palva & Palva, 2018). The significance of EEG ISA was demonstrated by the main occurrence of behaviors mainly at the specific oscillation phase of the EEG ISA (Fig. 2).

ISA can also be recorded by modalities other than EEG. Recently, the ISA of individual neurons was recorded by calcium imaging (Stringer et al., 2019) or electrophysiological unit recording (Cowley et al., 2020). These ISAs are associated with behaviors; the behaviors predict ISA (Stringer et al., 2019) or the ISA represents the impulsivity of behaviors (Cowley et al., 2020). However, further studies are needed to correlate these individual neuron ISAs with EEG ISAs.

The main limitation of the present study is that we did not investigate the mechanism by which EEG ISA modulates behaviors. We speculate that arousal is one such candidate; however, more mechanisms should be involved. We expect subsequent studies to investigate this.

This study revealed that several complex behaviors can be modulated by the oscillation phase of ISA of EEG. Our findings may be extended to investigations of relationships between various cognitive processes and ISA generatred from a number of distinct brain regions. Since the ocillation phase of ISA is related to the pattern of occurrence of certain behaviors, it would be plausible to use ISA to optimize efficient work environments in which those behaviors are involved. Further studies may follow to apply ISA as biofeedback to induce optimal behavioral performance.

## Conclusions

The present study aimed to address the following two questions regarding EEG ISA: (1) Do EEG ISAs in different brain regions modulate behavior differently? and (2) can EEG ISA modulation be extended to various types of behavior? In a previous study, Monto et al. (2008) showed that the behavioral performance of the somatosensory discrimination task was increased during the rising phase of EEG ISA measured at the Fz and Cz channels. In the present study, we extended the results of Monto et al. (2008) by using more complicated behaviors and inspecting a wider area of the brain. Specifically, we analyzed EEG ISA for six behaviors while playing a video game. We found that if the occurrence of a behavior (*e.g.*, crash into wall) did not depend on other behaviors (Fig. 1), the occurrence of this behavior was more strongly associated with the specific oscillation phase of EEG ISA (Fig. 2). This suggests that relatively simple perceptual behaviors and more diverse and complex behaviors can be modulated by EEG ISA. We also found that different behaviors were associated with different EEG ISA oscillation phases in different brain regions (Fig. 3). This suggests that the association of the EEG ISA with a certain behavior can be specific with respect to both the brain region and phase.

## Supplemental Information

10.7717/peerj.12875/supp-1 Supplemental Information 1 Dataset

10.7717/peerj.12875/supp-2 Supplemental Information 2 MATLAB code for loading raw data as MATLAB format

10.7717/peerj.12875/supp-3 Supplemental Information 3 MATLAB code for EEG processing

10.7717/peerj.12875/supp-4 Supplemental Information 4 MATLAB code for EEG analysis

## Additional Information and Declarations

### Data Availability

The following information was supplied regarding data availability: The code is available in the Supplemental Files, and the raw data is the publicly available dataset available at OpenNeuro: James F. Cavanagh and Joel Castellanos (2021). EEG: Continuous gameplay of an 8-bit style video game. OpenNeuro. [Dataset] doi: 10.18112/openneuro.ds003517.v1.1.0.
