# Supplementary material for: Differential modulation of behavior by infraslow activities of different brain regions
Source: PeerJ. 2022 Feb 1;10:e12875. doi: 10.7717/peerj.12875 (PMC8815366; doi:10.7717/peerj.12875)
Supplement: Supplemental Information 1 [file peerj-10-12875-s001.pdf]

Versions

1.0.0

2021-02-16

(/datasets/ds00351

1.1.0

2021-02-16

(/datasets/ds00351

EEG: Continuous gameplay of an 8-bit style video game

uploaded by James F Cavanagh on 2021-02-15 - 3 months ago
last modified on 2021-02-15 - 3 months ago
authored by James F Cavanagh, Joel Castellanos
14 2380

Valid 1 Warning

OpenNeuro Accession Number: ds003517

Files: 389, **Size:** 5.83GB, **Subjects:** 17, **Session:** 1

Tasks: ContinuousVideoGamePlay

Modalities: channels, coordsystem, eeg, electrodes, events

README

AUTHORS

DATASET DOI

10.18112/openneuro.ds003517.v1.1.0

LICENSE

ACKNOWLEDGEMENTS

HOW TO ACKNOWLEDGE

FUNDING

REFERENCES AND LINKS

ETHICS APPROVALS

HOW TO CITE

Text BibTeX Copy

James F Cavanagh and Joel Castellanos (2021). EEG: Continuous gameplay of an 8-bit style video game. OpenNeuro. [Dataset] doi: 10.18112/openneuro.ds003517.v1.1.0

More citation info (/cite)

COMMENTS

Please sign in to contribute to the discussion.
